# Commentary on: a review on delayed toxic effects of sulfur mustard in Iranian veterans

**DOI:** 10.1186/2008-2231-20-99

**Published:** 2012-12-26

**Authors:** Amir Shadboorestan

**Affiliations:** 1 Department of Toxicology and Pharmacology, Faculty of Pharmacy, Tehran University of Medical Sciences, Tehran, Iran

## 

Dear Editor-in-Chief

I read with interest the recent nice article published by Dr Razavi titled “A review on delayed toxic effects of sulfur mustard in Iranian veterans” [1]. Sulfur mustards (SM), commonly known as mustard gas are alkylating agents capable of causing short and long term morbidity [2].

The author stated in Table three that ophthalmic complications in Khateri et al. studies are 93.3% but there is a typesetting problem over there and the correct number is 39.3% [3]. The eyes are the most sensitive organ to SM exposure. Nearly 90% of eye injuries after SM exposure may cause later some kind of ocular disorders [4].

Also, in the paper of Razavi et al. [1], they have highlighted that SM can remain in the battlefields (for example beside the moats in World War I) and can be found in the amount of 1–25 mg/m3 in 6–12 inch depth of the soil. In moderate temperatures with mild winds, SM can remain stable for more than a week but some forms of SM can be stored in the soil for up to 10 years [1]. Natural degradation of SM in soil is a result of chemical hydrolysis and biodegradation. The major product of chemical hydrolysis is thiodiglycol. Chemical hydrolysis of SM and its chlorine derivatives in soil depends on soil type and moisture content, degree of contamination, and temperature. If the moisture content of soil is lower than 50% of its moisture capacity, then chemical hydrolysis in soil does not occur [5]. With higher temperatures and moisture content, the extent of hydrolysis of SM increases, but never to 100% completion. The SM is known to degrade faster in alkaline soils. If SM droplets are considerably below the soil surface, then SM can persist for several years [6,7]. The process of biodegradation of thiodiglycol is still longer [8]. Elimination, hydrolysis and sulfoxide products were also detected in extracts of soil samples from the Iran–Iraq war [9]. SM can be absorbed following inhalation [10] and dermal [10,11] exposure from air and soil. Therefore, although SM is a very reactive substance which hydrolyzing rapidly on contact with water, it may persist in the environment for many years, even decades, longer than expected [12].

Considering all the above facts, the correct assessment of the amount of agent in the air, in nearby water, on equipment, and on the ground (substrates such as soil, grass, concrete and asphalt) is critical to making correct decisions about the need for decontamination. Therefore, testing methods that detect both the SM and its degradation products, some of which may be toxic, since the war zones in our country now are major tourist areas where every year a large number of people travel there, are essential. Again, I would like to thank authors for collecting those valuable data using mostly domestic data and writhing such a nice review.
